# Iron nanoparticles mitigate cadmium-induced abiotic stress in soybean by modulating reactive oxygen species accumulation and cellular integrity

**DOI:** 10.3389/fpls.2025.1727507

**Published:** 2026-01-16

**Authors:** Ahmed Mukhtar, Shaista Jabeen, Muhammad Shoaib Asad, Muhammad Tauseef Jaffar, Mostafa A. Abdel-Maksoud, Salman Alrokayan, Xiaoli Chen, Xiaolong Ren

**Affiliations:** 1 College of Agronomy, Northwest A&F University, Yangling, China; 2 Key Laboratory of Crop Physiology, Ecology and Tillage in Northwest Loess Plateau, Minister of Agriculture, Yangling, China; 3 College of Life Sciences, Northwest A&F University, Yangling, China; 4 College of Natural Resources and Environment, Northwest A&F University, Yangling, China; 5 Research Chair of Biomedical Applications of Nanomaterials, Biochemistry Department, College of Science, King Saud University, Riyadh, Saudi Arabia

**Keywords:** abiotic stress, antioxidants, heavy metals, iron nanoparticles, photosynthesis, ROS fingerprints

## Abstract

Cadmium (Cd) is a toxic heavy metal that causes morphological and physiological changes to plants, which eventually leads to a decline in growth and productivity. Nanoparticle-based amelioration of Cd toxicity in plants is an environmentally friendly and cost-effective approach. Nevertheless, the interaction between iron nanoparticles (FeNPs) and Cd in soybean (*Glycine max* L.) is still not well documented. This research was conducted to evaluate the effects of soil application of FeNPs on soybean plants exposed to Cd stress. The size, surface morphology, and crystalline nature of the FeNPs were observed using scanning electron microscopy (SEM) and X-ray diffraction (XRD). Soil amended with FeNPs (50 mg L^−1^) decreased reactive oxygen species (ROS) accumulation and improved photosynthetic performance in comparison with Cd treatment (40 mg kg^−1^). These enhancements of peak fluorescence (Fp), maximum potential efficiency of photosystem II (Fv/Fm), photochemical quenching (Qp), and fluorescence decrease ratio (Rfd) were positively correlated with leaf stomatal opening and growth, while negatively correlated with non-photochemical quenching (NPQ), H_2_O_2_, and O_2_^·−^ content. Subcellular localization via transmission electron microscopy analysis showed that the FeNPs improved the intracellular integrity with the development of key cellular organelles and active Cd sequestration in plant vacuoles. Furthermore, FeNPs inhibited oxidative stress by 34%–56% via the modulation of antioxidant enzymes, i.e., superoxide dismutase (SOD), peroxidase (POD), glutathione (GSH), ascorbic acid (AsA), catalase (CAT), and reductase activity. Overall, FeNPs serve as a potent ameliorative agent against Cd-induced phytotoxicity by enhancing antioxidant defense mechanisms, optimizing photosynthetic performance, preserving cellular integrity, and significantly suppressing oxidative stress.

## 1 Introduction

The global prevalence of heavy metal contamination is exacerbated by a complex interplay of geogenic and climatic factors. In China, 402 industrial and 1,041 agricultural sites have been documented for the heavy metal pollution (Yang et al., 2018), caused by rapid urbanization, industrialization, and the enhancement of municipal, agricultural, and technological activities (Yu et al., 2025). High levels of arsenic (As), cadmium (Cd), mercury (Hg), and lead (Pb) have been majorly reported in the southwest, south coast, middle Yangtze River, east coast, northwest, north coast, middle Yellow River, and northeast (Huang et al., 2019). As a result of arising natural and anthropogenic activities, the agricultural soil of these specified regions has become more susceptible to the toxic heavy metals, including Pb, Cd, As, Cr, Cu, Hg, Ni, Al, and Zn (Hou et al., 2020). The persistent deposition of heavy metals in arable lands leads to soil toxicity levels that compromise plant growth and productivity, ultimately threatening agricultural sustainability (Wang et al., 2019).

Among all toxic metals, Cd poses significant environmental and health risks, as it is classified as a Group 1 human carcinogen (Liu et al., 2023). Cd toxicity inhibits seed germination and early seedling development (El Rasafi et al., 2020). Cd stress triggered a dose-dependent decrease in the content of chlorophyll of tobacco, where chlorophyll *a* and *b* reduced by 34.80% and 55.80%, respectively, under 50 mg L^−1^ Cd treatment (Yuan and Huang, 2016). Higher Cd levels (≥10 ppm) are associated with reduced growth (Reese and Roberts, 1984). Cd exhibits high phytotoxicity even at low concentrations, inducing plant growth retardation, physiological and metabolic disruptions, lipid peroxidation damage, and, ultimately, plant mortality (Huang et al., 2019). Therefore, effective strategies are required to minimize Cd accumulation and Cd-induced toxic impact on crops.

Soybean (*Glycine max* L.), a valuable protein and oil crop for human and animal consumption (Muhammad et al., 2024), has been documented for its capability to accumulate higher Cd content, which decreases its biomass, nitrogen content, and overall commercial value (Basal and Szabó, 2020; Cerezini et al., 2020). The highly mobile nature of Cd allows its maximum accumulation in edible parts of crops, pointing toward severe health complications (Wang et al., 2019; Cai et al., 2025), as reported by the highest Cd concentration in soy milk (Huang et al., 2025). The response of soybean grains to Cd contamination and health risks are more severe in South China than in North China (Zhang et al., 2021). Thus, heavy metal contamination of arable lands not only impairs plant growth and productivity but also poses a significant risk of air-, water-, and soil-mediated ultimate entries of toxic metals into the food chain. However, exploring sustainable strategies to mitigate heavy metal toxicity is essential for ensuring plant health and ecosystem sustainability.

Nanotechnology is emerging as a promising approach to enhance the detection, remediation, and mitigation of toxic metals like cadmium (Zhou et al., 2020). Carbon nanotubes (CNTs) possess an exceptionally high surface area, making them powerful adsorbents with a strong affinity for capturing cadmium ions (Rao et al., 2007). Zinc nanoparticles have been reported for their stimulatory role in improving nitrogen and carbon contents by 48% and 21%, respectively, in corn (Ahmad et al., 2025). Polymeric nanoparticles have significant potential to improve seed germination and water uptake under copper (Cu) stress (Xin et al., 2020). Titanium (TiO_2_) nanoparticles can adsorb metals and also use light energy to transform highly toxic metal species into less harmful forms (Kabra et al., 2004). Silver nanoparticles offer diverse approaches for enhancing plant tolerance and reducing metal accumulation (Jabeen et al., 2025). Iron nanoparticles have been remarkably highlighted for their significant potential to enhance the photosynthetic parameters, including chlorophyll *a* (50%), chlorophyll *b* (67%), total chlorophyll content (50%), and carotenoids (62%) (Zia-ur-Rehman et al., 2023), and to mitigate heavy metal stress by the effective alleviation of oxidative stress in wheat seedlings (Hu et al., 2007; Konate et al., 2017; Merinero et al., 2022). Moreover, iron nanoparticles significantly contribute to boost the morphophysiological attributes of strawberry (Mozafari et al., 2018) and soybean, specifically the nodule and shoot biomass by 99.0% and 55.4%, respectively, with different concentrations of 10 to 100 mg L^−1^ (Yang et al., 2020; Cao et al., 2022).

Despite the known impacts of Cd on soybean growth, the interactive effects of Cd and iron (Fe) supplementation, particularly through iron nanoparticles (FeNPs), remain poorly understood. Research is needed to elucidate the underlying mechanisms and optimal application strategies for FeNPs in Cd-stressed soybean. The objective of this study was to examine the impact of Cd on soybean growth measures, multiple photosynthetic efficiency parameters, reactive oxygen species (ROS) accumulation, and antioxidative responses under Cd alone and FeNP combined treatments. Another objective was to assess the cellular and subcellular variations by analyzing structural variation in stomata and key cellular organelles.

## 2 Materials and methods

### 2.1 Experimental design

The experiment was conducted in a growth chamber at Northwest A&F University, Yangling, Shaanxi, China. Soybean seeds were sterilized and germinated in seed propagation trays under a controlled temperature of 20°C during the night and 25°C during the day. Alongside this, soil spiking was conducted using CdCl_2_ as described in our previous work (Jabeen et al., 2025), and then pots (diameter, 18 cm; height, 20 cm) were filled according to the experiment design. Following the soil preparation, the uniformly sized seedlings were transplanted to the pots and maintained with watering.

The experiment comprised a completely randomized design (CRD) with three treatments, including control (CK), 40 mg kg^−1^ cadmium (Cd), and 40 mg kg^−1^ cadmium with 50 mg L^−1^ of iron nanoparticles (Cd + FeNPs). Every treatment was performed in three replicates, each having three plants.

### 2.2 Iron nanoparticle characterization and suspension preparation

Iron nanoparticles (Fe_3_O_4_) were purchased from Sigma-Aldrich (Merck, China). Prior to the experimental use of FeNPs, the characterization was performed to further confirm the reliability and efficiency based on their physicochemical properties. The surface morphology and size were examined via scanning electron microscopy (SEM), and the crystalline structure was assessed via X-ray diffraction (XRD). Afterward, a fresh FeNP suspension (50 mg L^−1^) was prepared each time using ultrasonication to avoid the agglomeration of particles. The prepared suspension was applied to the soil with an interval of 3 days in a total of seven applications to the FeNP-treated plants.

### 2.3 Photosynthetic efficiency analysis

The plants were covered with black cloth for approximately 1 hour, and then healthy leaves from each treatment were picked and positioned under a fluorometer (FluorCam, Germany) to analyze peak fluorescence (Fp), maximum potential efficiency of photosystem II (Fv/Fm), non-photochemical quenching (NPQ), photochemical quenching (Qp), and fluorescence decrease ratio (Rfd) (Jabeen et al., 2025).

### 2.4 Biomass assessment

Upon the completion of 110 days of germination, plants were harvested, followed by measurements of root and shoot fresh weight. Thereafter, both root and shoot samples were oven-dried at 60°C for 72 hours to measure the dry weight.

### 2.5 Histochemical detection of H_2_O_2_ and O_2_^·−^

To visualize ROS accumulation, 0.2% nitroblue tetrazolium (NBT) and 0.1% 3,3′-diaminobenzidine (DAB) solutions were prepared as described by Jabeen et al. (2025). Leaves were collected and directly placed in the prepared solutions, followed by infiltration for complete solution penetration, and stored in the dark for 10–12 hours. Subsequently, leaves were rinsed with ethanol until clear visualization of dark brown and blue spots, indicating DAB and NBT stains for H_2_O_2_ and O_2_^·−^ accumulation, respectively. Furthermore, to quantify H_2_O_2_, leaf extract was prepared with 0.1% trichloroacetic acid (TCA) as per the potassium iodide (KI) assay, and the absorbance was measured at 390 nm (Hussan et al., 2024a). O_2_^·−^ was calculated using the cytochrome *c* reduction method by preparing leaf extract with phosphate buffer and measuring the absorbance at 590 nm (Hussan et al., 2024b).

### 2.6 Enzymatic antioxidant activity

Fresh leaf sample (0.4 g) was homogenized in phosphate buffer and centrifuged (20 min, 4°C), and the crude enzyme extract was separated. The extract was used to measure enzyme activities.

#### 2.6.1 Superoxide dismutase

To measure superoxide dismutase (SOD) activity, 40 μL enzyme extract was used in the NBT photoreduction method with reaction illumination at 25°C and the absorbance measurements at 560 nm (Wu et al., 2024).

#### 2.6.2 Catalase

Catalase activity was measured by a preparation of a reaction mix of H_2_O_2_ (0.15 mL) and phosphate buffer (100 mL). The prepared reaction mix (2.9 mL) and crude enzyme extract (0.1 mL) were mixed, and the absorbance was recorded at 240 nm (Wu et al., 2024).

#### 2.6.3 Peroxidase

The reaction mix was prepared using guaiacol (28 μL) and phosphate buffer (50 mL) with mild heating, followed by the addition of H_2_O_2_ (19 μL) after completely normalizing at room temperature. Afterward, 3 mL of this reaction mix and 40 μL of crude enzyme extract were mixed, and the absorbance change was measured at 470 nm (Wu et al., 2024).

The observed SOD, peroxidase (POD), and catalase (CAT) rates were converted into units as per the following equation:


$$\text{SOD}\left|\text{POD}\right|\text{CAT activity}=\frac{\text{Units from cuvette}\times \text{Vt}}{\text{Vt}\times \text{W}}$$

Here, Vt is the total extracted volume, Vs is the volume used in the cuvette, and W is the leaf fresh weight.

### 2.7 Non-enzymatic antioxidant level

#### 2.7.1 Glutathione

Approximately 0.2 g of the leaf sample was homogenized and centrifuged at 1,200 rpm. The collected supernatant was mixed with 100 μL of sulfosalicylic acid and neutralized with 1.84 M triethanolamine. To quantify the glutathione (GSH) level, neutralized mix (50 μL) was added to nicotinamide adenine dinucleotide phosphate (NADPH) (20 μL), 5,5′-dithiobis(2-nitrobenzoic acid) (DTNB) (80 μL), and phosphate buffer (706 μL). The reaction was started by the addition of glutathione reductase (706 μL), and the absorbance was measured at 412 nm (Lou et al., 2018).

#### 2.7.2 Ascorbic acid

To quantify ascorbic acid (AsA) level, 0.2-g leaf samples were extracted in meta-phosphoric acid and centrifuged, and the obtained supernatant (0.3 mL) was mixed with phosphate-buffered saline (PBS) (0.75 mL) having ethylenediaminetetraacetic acid (EDTA) and dithiothreitol (DTT), followed by the addition of 0.6 mL of 10% TCA, 0.6 mL of 44% ortho-phosphoric acid, and α,α′-dipyridyl for color development. Afterward, 0.5 mL of 0.03% (w/v) FeCl_3_ was added, vortexed, and incubated for 30 min before measuring the absorbance at 525 nm (Lou et al., 2018).

#### 2.7.3 Flavonoids

Fresh leaf (0.5 g) samples were extracted with ice-cold 80% ethanol and centrifuged, and then the obtained supernatant (0.5 mL) was mixed with ethanol (1.5 mL), AlCl_3_ (0.1 mL), potassium acetate (0.1 mL), and water (mL). The prepared solution was incubated at room temperature for 30 min, and then the absorbance was recorded at 425 nm (Jabeen et al., 2023; Sutrisno and Kartini, 2025).

### 2.8 Leaf surface morphology under scanning electron microscopy

The fresh leaf samples were collected and placed immediately in glutaraldehyde. Afterward, the samples were processed via vacuum infiltration, then washed with PBS, and then sequentially dehydrated in 30%, 50%, 70%, 90%, and 100% ethanol. Subsequently, samples were dried with ethanol under a CO_2_ drying chamber and then visualized using a scanning electron microscope (Nano SEM, USA) (Jabeen et al., 2025).

### 2.9 Ultrastructural assessment under transmission electron microscopy

The small leaf sections were cut, placed in fixative, and then dried in a series of ethanol concentrations. Subsequently, samples were processed with LR White and dried in a hot air oven, and thin slices were cut to visualize changes at the subcellular level. These thin slices were stained prior to visualization using a transmission electron microscope (TEM) (High-Technologies Corporation, Japan) (Jabeen et al., 2025).

### 2.10 Statistical analysis and visualization

The data were analyzed and graphed on Origin version 2025b. Tukey’s test was used for means comparison. To assess the association between plant growth, antioxidants, photosynthesis, and reactive oxygen species (H_2_O_2_ and O_2_^·−^), Pearson’s correlation and the Mantel test were applied using RStudio version 2025.09.1 + 401. The systematic diagram of Cd-induced toxicity and the migration role of FeNPs was created on CNS*Knowall.com* (CNSknowall, 2025).

## 3 Results

### 3.1 Characterization of iron nanoparticles

**Figure 1:**
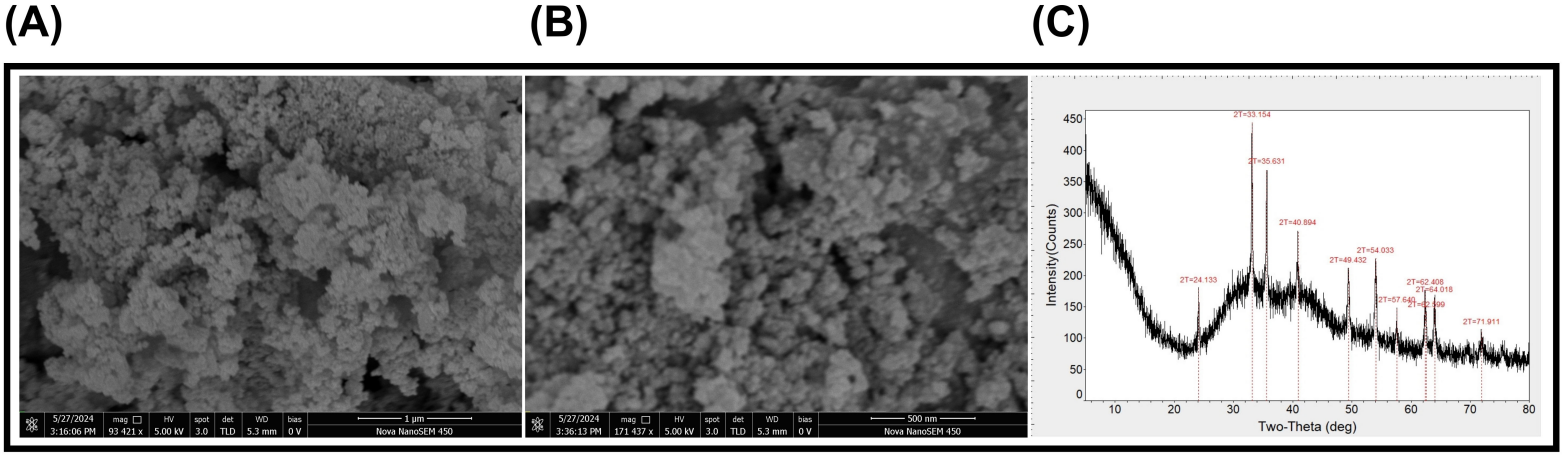
Characterization of iron nanoparticles (Fe_2_O_3_); **(a)** SEM (scale bar = 1 μm), **(b)** SEM (scale bar = 500 nm), and **(c)** XRD spectrum. SEM, scanning electron microscopy; XRD, X-ray diffraction.

Scanning electron micrographs revealed the uniform distribution and spherical shape of FeNPs (**Figures 1A, B**). The XRD analysis presented different diffraction peaks at 2θ = 24.13°, 33.15°, 35.63°, 40.89°, 49.43°, 54.03°, 57.64°, 62.40°, 62.59°, 64.01°, and 71.91° (**Figure 1C**). These multiple sharp peaks clarify the phase purity and highly crystalline nature of FeNPs. Moreover, full width at half maximum values (0.116–0.525) demonstrate well-ordered arrangements and nano-sized crystallite domains as mentioned in the **Supplementary Material**. Overall, the crystalline structure, high reactivity, and surface area of FeNPs suggest their stability for strong and effective interaction with soil and plant tissues.

### 3.2 Chlorophyll fluorescence indicators

**Figure 2:**
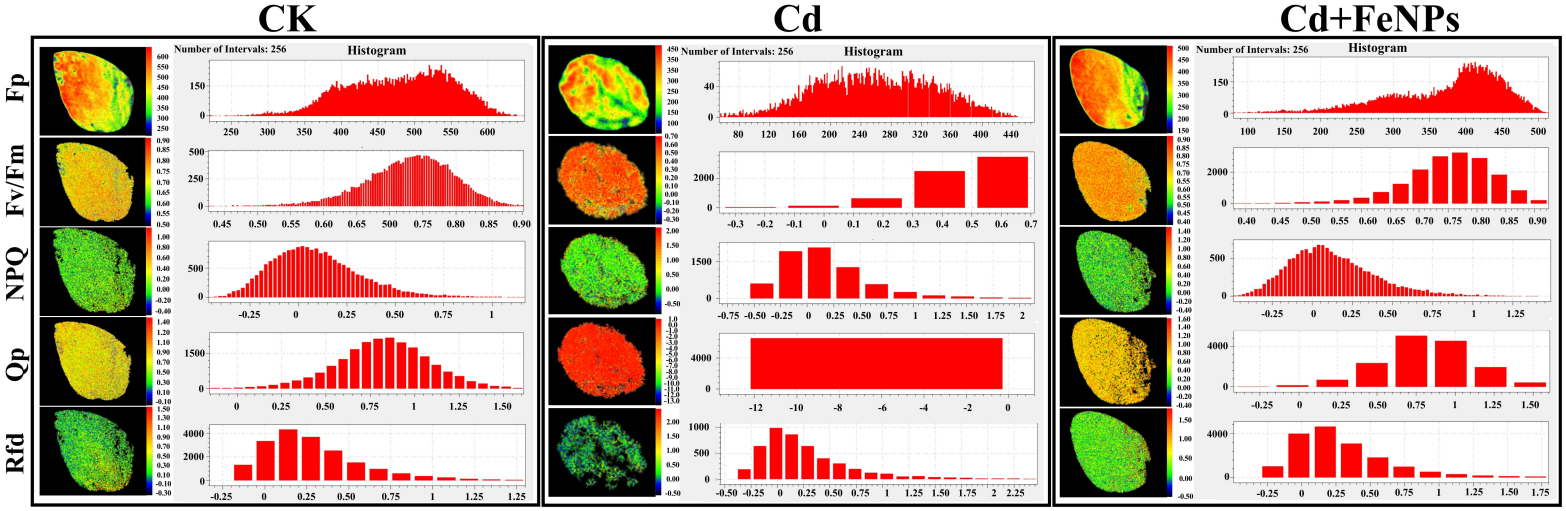
Chlorophyll fluorescence efficiency measures of soybean under control (CK), cadmium (40 mg kg^−1^ Cd), and cadmium + iron nanoparticles (40 mg kg^−1^ Cd + 50 mg L^−1^ FeNPs). Fp, peak fluorescence; Fv, variable fluorescence; Fm, maximum fluorescence; NPQ, non-photochemical quenching; Qp, photochemical quenching coefficient; Rfd, fluorescence decrease ratio (Rfd); FeNPs, iron nanoparticles.

Under CK, the Fp level, Fv/Fm, Qp, and Rfd were 477, 0.75, 0.82, and 0.31, respectively. The Cd exposure significantly decreased Fp to 259, Fv/Fm to 0.45, Qp to 0.57, and Rfd to 0.19. However, FeNP application recovered the photosystems and increased Fp, FV/Fm, Qp, and Rfd to 382, 0.76, 0.8, and 0.22, respectively. Similar to these photosynthetic measures, NPQ also showed Cd-induced stress by the enhancement of NPQ value from 0.10 to 0.26, while FeNPs decreased it to 0.14 (**Figure 2**). Overall, Cd impaired the chlorophyll fluorescence efficiency of photosystem II (PSII), but FeNPs limited the Cd-induced damage and partially restored the plant toward normal photosynthetic activity.

### 3.3 Plant biomass regulation

**Figure 3:**
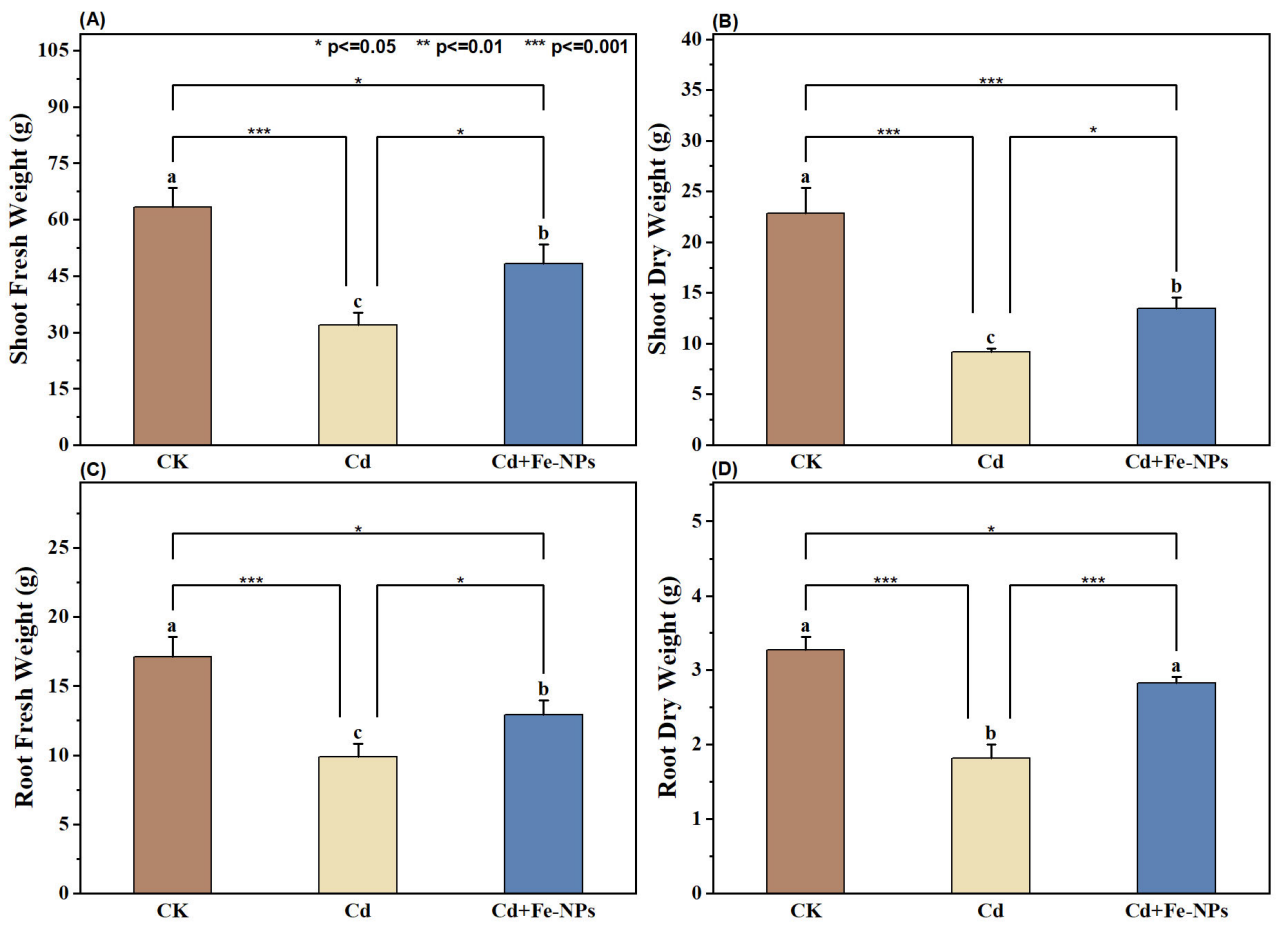
Shoot **(A, B)** and root **(C, D)** biomass of soybean under control (CK), cadmium (40 mg kg^−1^ Cd), and cadmium + iron nanoparticles (40 mg kg^−1^ Cd + 50 mg L^−1^ FeNPs). Tukey’s test was used for means comparison. The bar represents mean values, and the letters above bars represent the SE (n = 3). Asterisks above bars indicate the statistically significant differences among treatments. FeNPs, iron nanoparticles.

Cd induced a remarkable decline (47%–66%) in all observed growth measures under Cd stress, when compared with the CK group. Soil application of FeNPs resulted in increased shoot fresh weight (SFW; +54%), root fresh weight (RFW; +33%), shoot dry weight (SDW; +44%), and root dry weight (RDW; +100%). In general, FeNPs have significant potential to mitigate Cd-induced growth retardation and to boost the metabolic activity of soybean (**Figure 3**).

### 3.4 Histochemical and quantitative assessment of ROS

**Figure 4:**
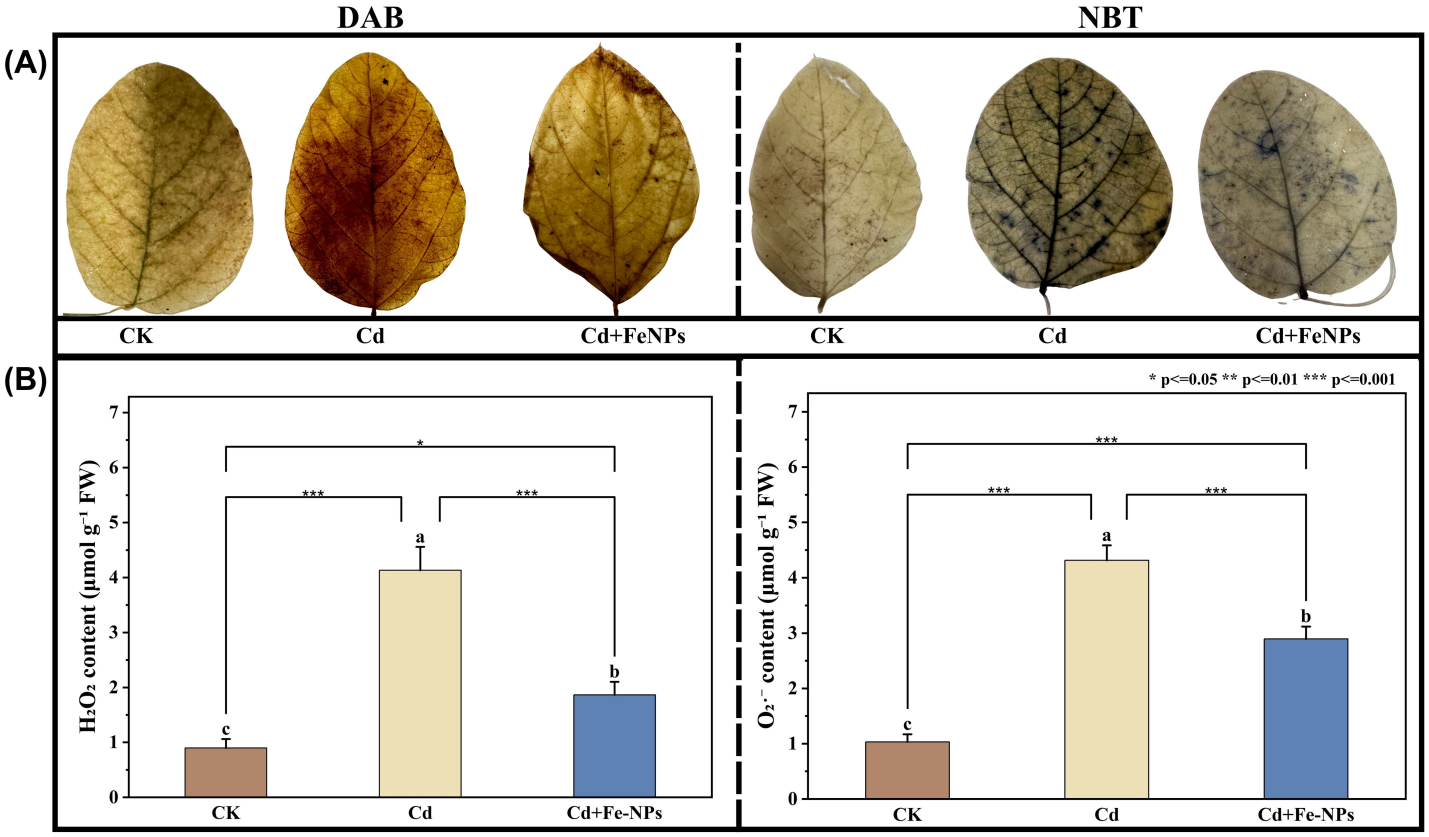
Reactive oxygen species (ROS) accumulation in the soybean leaf under control (CK), cadmium (40 mg kg^−1^ Cd), and cadmium + iron nanoparticles (40 mg kg^−1^ Cd + 50 mg L^−1^ FeNPs). 3,3′-Diaminobenzidine (DAB) and nitroblue tetrazolium (NBT) are used for *in situ* detection of hydrogen peroxide (H_2_O_2_) and superoxide radicals (O_2_^·−^). **(A)** Histochemical staining. **(B)** Quantification analysis. Tukey’s test was used for means comparison. The bar represents mean values, and the letters above bars represent the SE (n = 3). Asterisks above bars indicate the statistically significant differences among treatments. FeNPs, iron nanoparticles.

ROS accumulation was assessed using a quantitative method and histochemical staining. Under Cd stress, quantitative analysis showed a remarkable increase in ROS (**Figure 4B**), while DAB and NBT staining further validated the significant number of dark brown and blue spots (**Figure 4A**), respectively, indicating the higher accumulation of H_2_O_2_ and O_2_^·−^. FeNP supplementation to the Cd-stressed plants minimized the staining intensity and ROS quantification (34%–56%), which clarified the FeNPs’ potential to effectively minimize the Cd-induced oxidative stress (**Figure 4**).

### 3.5 Antioxidant responses

**Figure 5:**
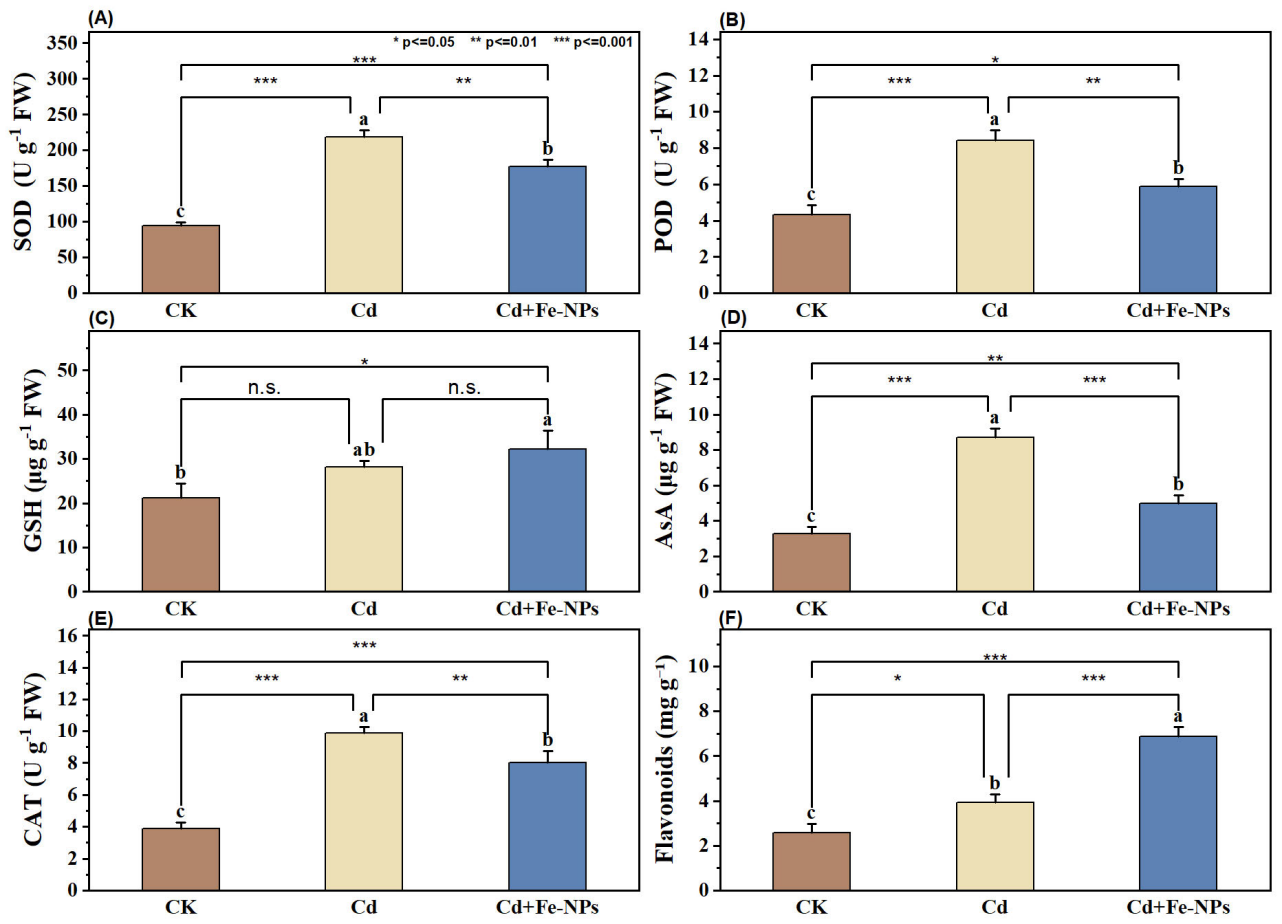
Enzymatic and non-enzymatic antioxidant response in soybean under control (CK), cadmium (40 mg kg^−1^ Cd), and cadmium + iron nanoparticles (40 mg kg^−1^ Cd + 50 mg L^−1^ FeNPs) on **(A)** superoxide dismutase (SOD), **(B)** peroxidase (POD), **(C)** reduced glutathione (GSH), **(D)** ascorbic acid (AsA), **(E)** catalase (CAT), and **(F)** flavonoids. Statistics: Tukey’s test was used for means comparison. The bar represents mean values, and the letters above bars represent the SE (n = 3). Asterisks above bars indicate the statistically significant differences among treatments. FeNPs, iron nanoparticles.

Cd alone and FeNP combined treatments showed variable responses to antioxidant activities. All enzymatic and non-enzymatic antioxidants increased under both Cd alone and FeNP combined treatments, indicating the active defensive mechanism against Cd stress. Maximum upsurge in AsA (168%), CAT (154%), SOD (132%), and POD (95%) was observed under Cd stress. Flavonoid (172%) and GSH (52%) exhibited maximum increase under the combined treatment of Cd and FeNPs, when compared with the CK (**Figure 5**). In general, antioxidant activity was markedly elevated under Cd stress; however, FeNPs mitigated oxidative stress, thereby reducing the need for antioxidant activation.

### 3.6 Stomatal aperture under scanning electron microscopy

**Figure 6:**
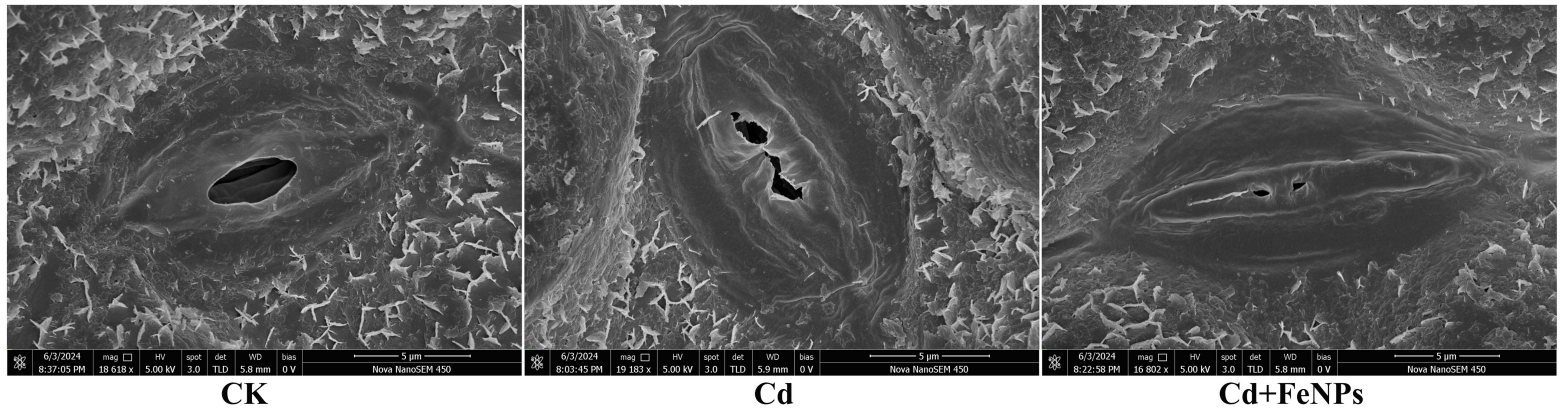
The scanning electron micrographs of soybean under control (CK), cadmium (40 mg kg^−1^ Cd), and cadmium + iron nanoparticles (40 mg kg^−1^ Cd + 50 mg L^−1^ FeNPs). FeNPs, iron nanoparticles.

Scanning electron micrographs showed a similar waxy leaf surface but variant stomatal opening and morphology among all treatments. Control plants revealed wider and smoother stomatal apertures, while Cd-stressed plants exhibited deformed guard cells and partially to totally closed stomata. However, FeNPs presented comparatively improved stomatal structure and enhanced aperture length, hence indicating the defensive role of FeNPs in leaf structural integrity against Cd stress (**Figure 6**).

### 3.7 Ultrastructural alterations

**Figure 7:**
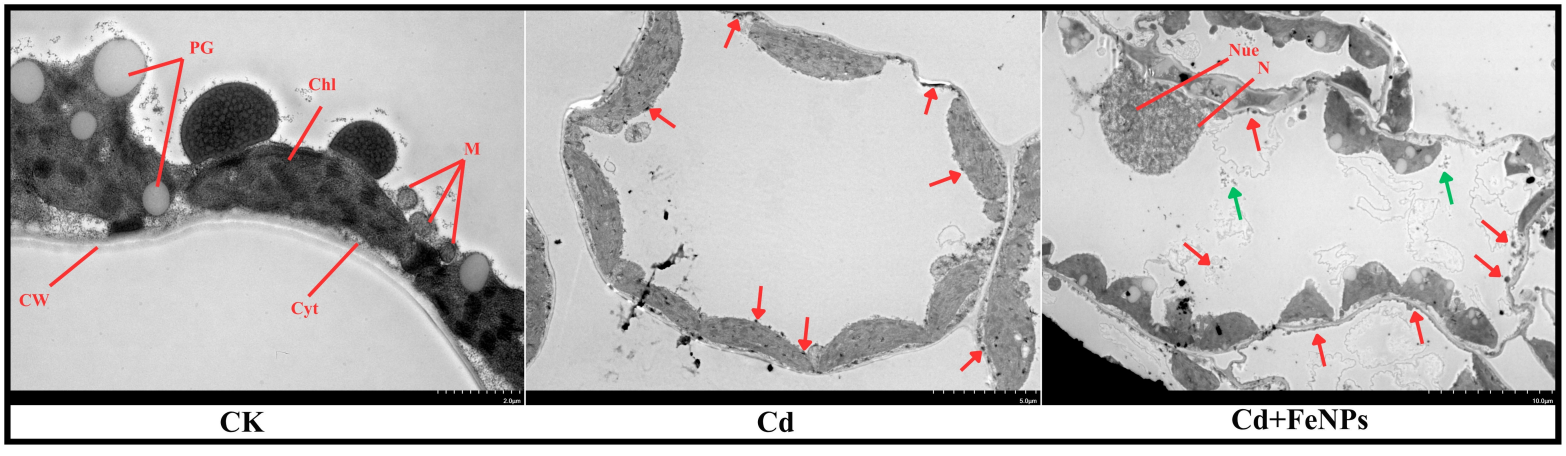
The transmission electron micrographs of soybean leaf from control (CK), cadmium (40 mg kg^−1^ Cd), and cadmium + iron nanoparticles (40 mg kg^−1^ Cd + 50 mg L^−1^ FeNPs). CW, cell wall; Cyt, cytoplasm; PG, plastoglobules; Chl, chloroplast; M, mitochondria; N, nucleus; Nue, nucleolus. Red and green arrows indicate Cd and FeNPs, respectively. FeNPs, iron nanoparticles.

Transmission electron micrographs presented well-developed, spherical-shaped cells with multiple chloroplasts and mitochondria, while Cd-induced damage deformed the key organelles with massive accumulation of cadmium alongside the cell wall and chloroplast (**Figure 7**). However, the combined application of FeNPs with Cd revealed substantial recovery by the development of nucleus, dense chloroplasts, and mitochondria, heightening the FeNPs’ protective function in preserving cellular ultrastructure (**Figure 7**).

### 3.8 Pearson’s and Mantel correlation

**Figure 8:**
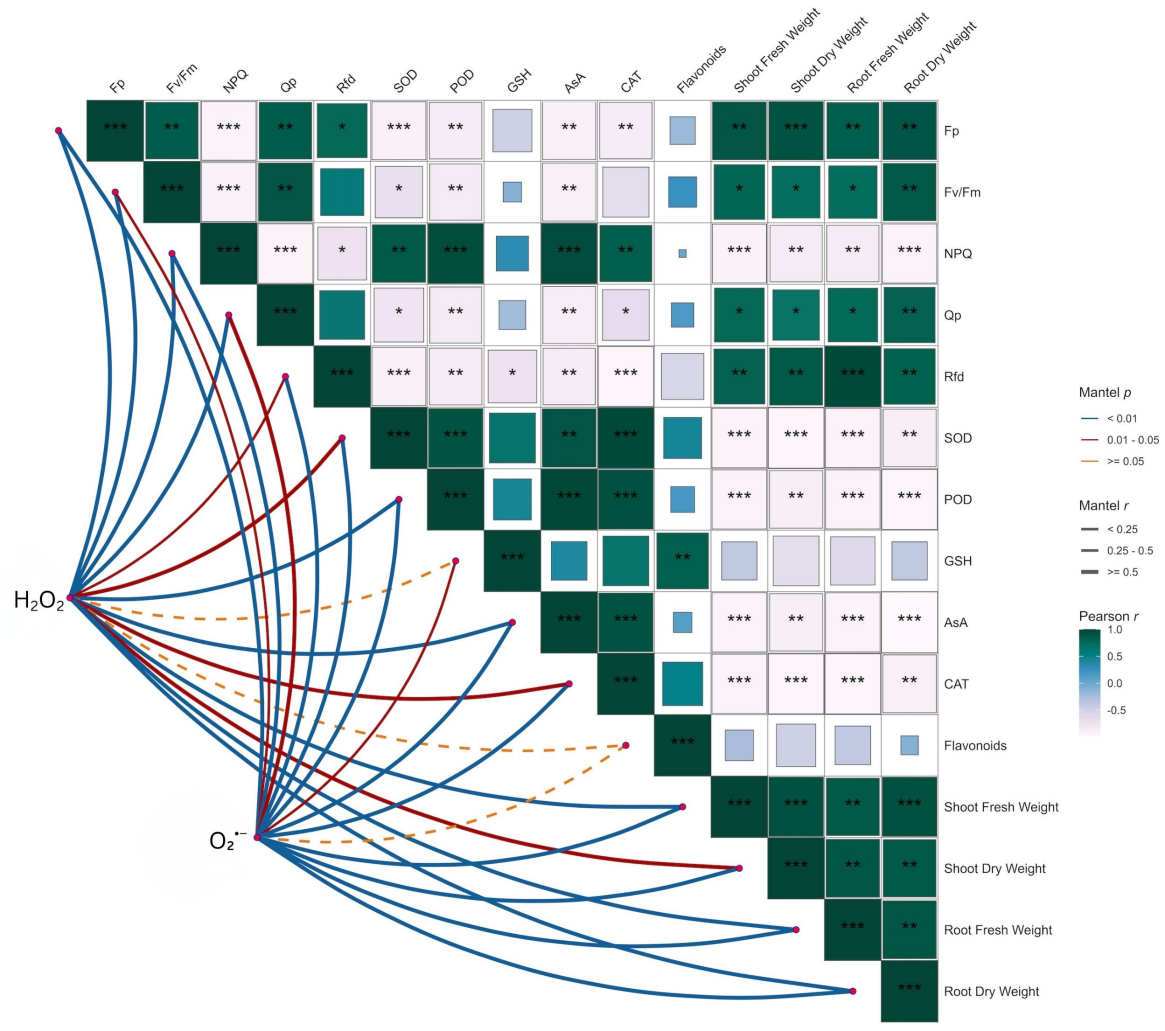
Pearson’s and Mantel correlation between soybean growth, photosynthesis, antioxidants, and ROS accumulation. The color gradient represents the strength and direction of Pearson’s correlation coefficient (r), while the symbols (*, **, and ***) indicate the statistical significance (p < 0.05, p < 0.01, and p < 0.001, respectively) of those correlations. ROS, reactive oxygen species.

According to Pearson’s analysis, photosynthetic indices (Fp, Fv/Fm, Qp, and Rfd), flavonoids, and biomass (shoot/root fresh and dry weight) show moderate to strong positive relationships (r ≥ 0.5; mostly p < 0.01) among themselves. Reactive oxygen species (H_2_O_2_, O_2_^·−^) negatively correlate with photosynthesis and biomass but positively correlate with antioxidants (SOD, POD, CAT, GSH, and AsA), showing the stress-induced activation of defense mechanisms. The antioxidant compounds intercorrelate and are closely related to flavonoids. Mantel tests support these trends: strong and significant couplings (solid/blue, thicker arcs; p < 0.01) between ROS and antioxidant matrices, moderate associations with photosynthetic traits, and weaker or non-significant links (dashed/orange) with biomass (**Figure 8**). In general, strong photosynthesis is associated with more growth, while high ROS inhibits growth and stimulates antioxidant defenses.

## 4 Discussion

Heavy metals, specifically cadmium, pose a significant threat to the food chain and food security (Deng et al., 2025; Shetty et al., 2025; Zhao et al., 2025). Nanotechnology offers new strategies for eco-friendly crop production, thereby providing alternative approaches to counteract the threats of abiotic stress, such as heavy metal stress in agricultural crops (Kaleem et al., 2025; Wang et al., 2025; Younas et al., 2025). FeNPs encompass considerable potential for alleviating heavy metal toxicity by stimulating nutrient uptake, photosynthetic activity, and antioxidant mechanisms (He et al., 2025a). The uptake and efficiency of nanoparticles (NPs) in plants largely depend on the shape, size, surface charge, and crystalline nature of nanoparticles (Elsayed et al., 2025; Jabeen et al., 2025). The method of nanoparticle application is also an important factor to ensure the effectiveness of nanoparticles; for example, soil application of nanoscale zero-valent iron (nZVI) can accelerate Cd immobilization and can remove Cd by physical sorption, chemisorption, or surface complex formation (Huang et al., 2021). Moreover, nanoparticles’ effectiveness can also be altered on the basis of their physicochemical properties, application dose, plant species, and plant growth conditions (Yasin et al., 2024; Gam et al., 2025; He et al., 2025a).

In this study, soybean growth was highly inhibited by Cd toxicity, as we observed a prominent reduction in plant biomass, including both shoot and root fresh and dry weight (**Figure 3**). These are in line with the results of the previous study on tobacco (Jabeen et al., 2025) and alfalfa (Hussan et al., 2024a). This growth retardation is a commonly described effect of Cd, which acts to disorganize nutrient and water assimilation, suppress metabolic determinants, and cause direct cell damage. Prior to experimental implication of FeNPs, we confirmed their effectiveness by assessing the minimal size, spherical shape, and crystalline nature (**Figure 1**). The soil supply of FeNPs mitigated Cd toxic effects on soybean (*G. max* L.) growth and photosynthetic efficiency (**Figures 2**, **3**) and improved the antioxidant activity and cellular ultrastructure under Cd stress. Previous studies have shown that FeNPs increased nutrient availability to Cd toxicity in rice (Zhou et al., 2023) and lima bean (Ahmad et al., 2021).

Cd-mediated plant stress is mainly caused by disruptions in the iron homeostasis, as Cd mimics iron and competes for uptake, which leads to iron deficiency. This decrease in iron impairs iron-dependent mechanisms, including photosynthesis and several enzymatic processes, and enhances ROS accumulation (Luo et al., 2024). As a result, plants activate their defensive responses, such as the activation of the phenyl propanoid pathway and the synthesis of flavonols (Cao et al., 2024), SOD, POD, and CAT (He et al., 2025b). The FeNPs effectively immobilize Cd, gradually lower the iron deficiency, strengthen the metabolic processes (Ainiwaer et al., 2024), and assist plants to boost their antioxidative activity (Lee et al., 2023). Overall, FeNPs have significant potential to alleviate Cd toxicity.

**Figure 9:**
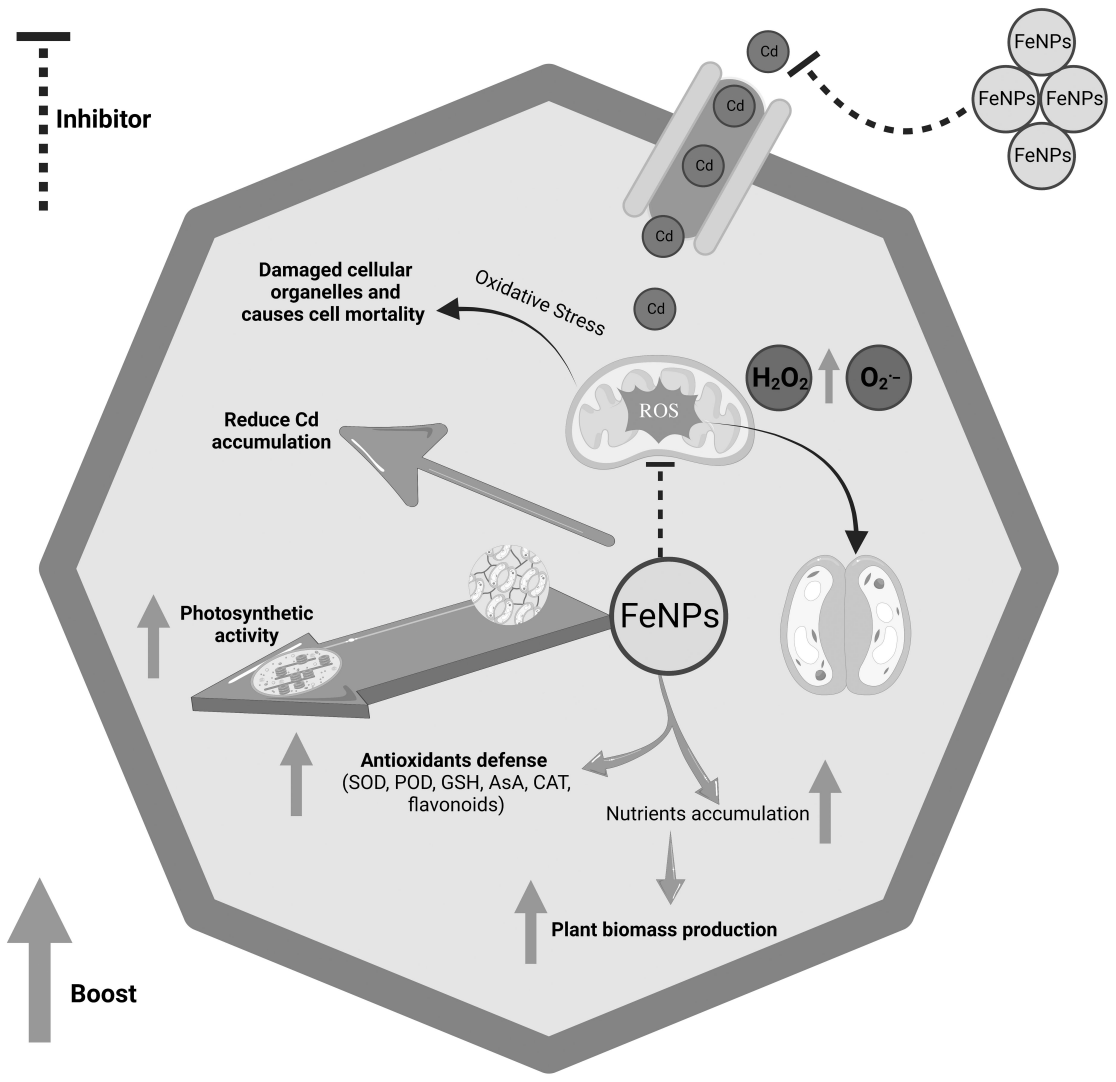
Systematic diagram of Cd-induced toxicity and migration role of FeNPs. Upward green arrows show the increase in attributes. FeNPs, iron nanoparticles.

In this study, we observed a substantial recovery of plant biomass by supplementation of FeNPs to Cd-stressed plants. This may be due to the dual function of iron as a micronutrient and a modulator of stress. Iron is the cofactor for enzymes of chlorophyll synthesis and cellular respiration, while supplementation of FeNPs may have enhanced these basic processes, thus assisting the general plant growth by stimulating antioxidant defense (**Figure 9**).

A major causative factor for this growth inhibition is the reduction of photosynthesis. In the current work, a significant reduction occurred in the chlorophyll fluorescence parameters (Fp, Fv/Fm, QP, NPQ, and Rfd) of Cd-stressed plants in soybean (**Figure 2**). This resulted from Cd interfering with the photosynthetic electron transport system and inhibiting the plant from fixing carbon as well as energy. The loss of photosynthetic apparatus destroys chloroplasts and interferes with the production of chlorophyll, consequently decreasing carbon fixation. This stress can decrease the Fv/Fm ratio, which reflects that the photosynthesis rate is slow and electron transferring ability is weak; finally, essential pigments are destroyed (Salam et al., 2022; Ghouri et al., 2023). This injury is augmented with morphological alterations to the leaf surface. We observed under SEM that Cd exposure resulted in conspicuous stomatal closure and distortion of guard cells (**Figure 6**). Stomata play an important role in inducing uptake for photosynthesis, and their closing under Cd stress greatly reduces the availability of intracellular CO_2_ that, in turn, decreases the photosynthetic rate (Wang et al., 2022; De Souza, 2023). This combined attack on the biochemical machinery of PSII and the physical passage for gas exchange accounts for the dramatic effect of Cd on soybean photosynthesis.

The reduction of oxidative stress is the central route to recovery. Cd was reported to enhance the overproduction of ROS, such as hydrogen peroxide (H_2_O_2_) and superoxide radical (O_2_^·−^), by lipid peroxidation, which extensively induces damage in cells. Our findings revealed that the application of FeNPs had a negative impact on ROS accumulation under Cd stress in soybean tissues (**Figure 4**). This was brought about by enhancing the plant’s endogenous antioxidant defense system. We observed increased activities of the major antioxidant enzymes such as SOD, POD, and CAT along with elevated non-enzymatic antioxidants such as reduced GSH and AsA; similar results were noticed in wheat (Konate et al., 2017). SOD provides a primary defense mechanism by transforming O_2_^·−^ into H_2_O_2_, which is subsequently detoxified into water and oxygen by CAT and POD (Hussain et al., 2019; Long et al., 2024).

Systemic protection at the cellular level was seen from TEM images. Under Cd stress, soybean cells exhibited massive accumulation of Cd around the cell wall (**Figure 7**). Similarly, barley under Cd exposure of 300 mg/kg showed granular Cd deposits around the cell wall and tonoplast (Ma et al., 2023). The electron micrographs of leaves and pericycle cells of *Dittrichiaviscosa* showed Cd deposits by supplementation of Cd (100 mg L^−1^) (Fernández et al., 2014). The *Allium sativum* roots also showed electron-dense granules in vacuoles and nucleoli under 10 mM Cd supplementation (Liu and Kottke, 2003). *Arabidopsis thaliana* showed Cd deposits in the endodermis, symplast, and intercellular space of cortex after 50- and 5-µm Cd exposure (Van Belleghem et al., 2007). Another research also focused on Cd subcellular localization, and they highlighted black deposits in electron micrographs of root (cross-section) and leaf (vertical section) by the application of 500 μM Cd^2+^ in 10% Hoagland solution for 1 week (Lan et al., 2019). This Cd accumulation induced ultrastructural destruction, such as ruptured membranes of cell membranes, disorganized cytoplasm, and swollen or dissolved mitochondria. In contrast, plant cells treated with FeNPs had complete cell structures, such as well-shaped organelles, firm cell wall boundaries, and clear nuclear membranes. This maintenance of cell architecture is a direct consequence of the mitigated oxidative stress and highlights the remarkable protection potential that FeNPs can provide at the subcellular level (Jabeen et al., 2025). In addition, the stomatal closure induced by Cd in SEM was suppressed following FeNP treatment, thereby allowing normal gas exchange, and it could be concluded that it was responsible for the recovery of photosynthetic rate and overall vigor of the plant.

## 5 Conclusion

In summary, our study indicates that Cd stress significantly inhibits the growth of soybean through oxidative damage, the destruction of photosynthetic systems, and ultrastructural alteration at the subcellular level, mainly due to Cd accumulation. FeNPs have effectively alleviated Cd-induced cytotoxic effects by reducing Cd accumulation, improving the antioxidant defense system of the plant, maintaining the integrity of cellular organelles, and restoring photosynthesis. Collectively, these results highlight the potential of iron nanoparticles as a promising strategy to enhance crop tolerance and support food security in Cd-contaminated agricultural soils.

## Data Availability

The original contributions presented in the study are included in the article/**Supplementary Material**. Further inquiries can be directed to the corresponding authors.

## References

[B2] Ahmad W. Nepal J. Xin X. Nadeem M. He Z. (2025). Nano zinc oxide enhances corn growth and nutrient uptake: comparison between soil drench and seed coating applications in alkaline sandy soils. J. Soil Sci. Plant Nutr. 25, 1–13. doi: 10.1007/s42729-025-02329-8

[B1] Ahmad A. Yasin N. A. Khan W. U. Akram W. Wang R. Shah A. A. . (2021). Silicon assisted ameliorative effects of iron nanoparticles against cadmium stress: attaining new equilibrium among physiochemical parameters, antioxidative machinery, and osmoregulators of Phaseolus lunatus. Plant Physiol. Biochem. 166, 874–886. doi: 10.1016/j.plaphy.2021.06.016, PMID: 34237605

[B3] Ainiwaer M. Jia H. Zhang T. Huang J. Zhang N. Yin X. . (2024). Effective co-immobilization of arsenic and cadmium in contaminated soil by sepiolite-modified nano-zero-valent iron and its impact on the soil bacterial community. Sci. Rep. 14, 26178. doi: 10.1038/s41598-024-77066-6, PMID: 39478164 PMC11525559

[B4] Basal O. Szabó A. (2020). Physiomorphology of soybean as affected by drought stress and nitrogen application. Scientifica 2020, 6093836. doi: 10.1155/2020/6093836, PMID: 32351758 PMC7171675

[B5] Cai L.-M. Wang A. He S. Fan Y.-M. Wen H.-H. Luo J. . (2025). Determination of site-specific natural background values for soil potentially toxic elements supported by partial least squares regression method. J. Hazardous Materials 498, 139911. doi: 10.1016/j.jhazmat.2025.139911, PMID: 40974678

[B7] Cao Y. Mei Y. Zhang R. Zhong Z. Yang X. Xu C. . (2024). Transcriptional regulation of flavonol biosynthesis in plants. Horticulture Res. 11, uhae043. doi: 10.1093/hr/uhae043, PMID: 38623072 PMC11017525

[B6] Cao X. Yue L. Wang C. Luo X. Zhang C. Zhao X. . (2022). Foliar application with iron oxide nanomaterials stimulate nitrogen fixation, yield, and nutritional quality of soybean. ACS Nano 16, 1170–1181. doi: 10.1021/acsnano.1c08977, PMID: 35023717

[B8] Cerezini P. Kuwano B. H. Grunvald A. K. Hungria M. Nogueira M. A. (2020). Soybean tolerance to drought depends on the associated Bradyrhizobium strain. Braz. J. Microbiol. 51, 1977–1986. doi: 10.1007/s42770-020-00375-1, PMID: 32918241 PMC7688821

[B9] CNSknowall (2025). Mechanism diagram. Available online at: https://cnsknowall.com (Accessed October, 20, 2025).

[B11] Deng P. Hu X. Mu L. Yu F. Luo L. (2025). Application of a machine learning-based food risk framework to assess the public dietary risk of cadmium in China. Environ. pollut. 384, 126911. doi: 10.1016/j.envpol.2025.126911, PMID: 40744370

[B10] De Souza A. P. (2023). Dynamic responses of carbon assimilation and stomatal conductance in the future climate. J. Exp. Bot. 74, 2790–2793. doi: 10.1093/jxb/erad049, PMID: 37103002

[B12] El Rasafi T. Bouda S. Nouri M. Haddioui A. (2020). Assessment of metals (Cu, Ni) and metalloids (As) induced stress responses in Barley (Hordeum vulgare) and wheat (Triticum aestivum). J. Mater Environ. Sci. 11, 795–807.

[B13] Elsayed M. E. Ayoub H. A. Helal M. I. Sang W. Shen Z. Abdelhafeez I. A. (2025). Nanotechnology-enabled soil management for sustainable agriculture: interactions, challenges, and prospects. Environ. Science: Nano 12, 2128–2153. doi: 10.1039/D4EN00943F

[B14] Fernández R. Fernández-Fuego D. Bertrand A. González A. (2014). Strategies for Cd accumulation in Dittrichia viscosa (L.) Greuter: role of the cell wall, non-protein thiols and organic acids. Plant Physiol. Biochem. 78, 63–70. doi: 10.1016/j.plaphy.2014.02.021, PMID: 24636908

[B15] Gam H.-J. Woo J.-I. Injamum-Ul-Hoque M. Ahsan S. Imran S. Sarker A. . (2025). Unlocking key factors and mechanistic insight of cadmium toxicity mitigation using green-synthesized ZnO nanoparticles in soybean through advanced metabolomics. Environ. Technol. Innovation 40, 104422. doi: 10.1016/j.eti.2025.104422

[B16] Ghouri F. Shahid M. J. Liu J. Lai M. Sun L. Wu J. . (2023). Polyploidy and zinc oxide nanoparticles alleviated Cd toxicity in rice by modulating oxidative stress and expression levels of sucrose and metal-transporter genes. J. Hazardous Materials 448, 130991. doi: 10.1016/j.jhazmat.2023.130991, PMID: 36860085

[B18] He T. Hao X. Chen Y. Li Z. Zheng X. Yang M. . (2025b). Promoting the growth of rice and reducing the accumulation of Cd in rice by pig bedding derived carbon dots (PBCDs) under Cd stress. Environ. Science: Nano 12, 863–878. doi: 10.1039/D4EN00682H

[B17] He D. Kaleem Z. Ali S. Shahbaz H. Zhang K. Li J. . (2025a). Impact of iron oxide nanoparticles on cadmium toxicity mitigation in Brassica napus. Plant Physiol. Biochem. 220, 109500. doi: 10.1016/j.plaphy.2025.109500, PMID: 39813760

[B19] Hou D. O’Connor D. Igalavithana A. D. Alessi D. S. Luo J. Tsang D. C. . (2020). Metal contamination and bioremediation of agricultural soils for food safety and sustainability. Nat. Rev. Earth Environ. 1, 366–381. doi: 10.1038/s43017-020-0061-y

[B20] Hu J. Lo I. M. Chen G. (2007). Comparative study of various magnetic nanoparticles for Cr (VI) removal. Separation Purification Technol. 56, 249–256. doi: 10.1016/j.seppur.2007.02.009

[B22] Huang N. Wang B. Liu S. Wang K. Wang R. Liu F. . (2025). Cadmium exposure in infants and children: Toxicity, health effects, dietary risk assessment and mitigation strategies. Crit. Rev. Food Sci. Nutr. 65, 5085–5107. doi: 10.1080/10408398.2024.2403036, PMID: 39264340

[B23] Huang Y. Wang L. Wang W. Li T. He Z. Yang X. (2019). Current status of agricultural soil pollution by heavy metals in China: A meta-analysis. Sci. Total Environ. 651, 3034–3042. doi: 10.1016/j.scitotenv.2018.10.185, PMID: 30463153

[B21] Huang D. Yang Y. Deng R. Gong X. Zhou W. Chen S. . (2021). Remediation of Cd-contaminated soil by modified nanoscale zero-valent iron: role of plant root exudates and inner mechanisms. Int. J. Environ. Res. Public Health 18, 5887. doi: 10.3390/ijerph18115887, PMID: 34070880 PMC8197846

[B24] Hussain A. Ali S. Rizwan M. ur Rehman M. Z. Qayyum M. F. Wang H. . (2019). Responses of wheat (Triticum aestivum) plants grown in a Cd contaminated soil to the application of iron oxide nanoparticles. Ecotoxicology Environ. Saf. 173, 156–164. doi: 10.1016/j.ecoenv.2019.01.118, PMID: 30771659

[B25] Hussan M. U. Hussain S. Adeel M. Ayub A. Kareem H. A. Jabeen S. . (2024a). Calcium oxide nanoparticles ameliorate cadmium toxicity in alfalfa seedlings by depriving its bioaccumulation, enhancing photosystem II functionality and antioxidant gene expression. Sci. Total Environ. 955, 176797. doi: 10.1016/j.scitotenv.2024.176797, PMID: 39395484

[B26] Hussan M. U. Hussain S. Hafeez M. B. Ahmed S. Hassan M. U. Jabeen S. . (2024b). Comparative role of calcium oxide nanoparticles and calcium bulk fertilizer to alleviate cadmium toxicity by modulating oxidative stress, photosynthetic performance and antioxidant-defense genes expression in alfalfa. Plant Physiol. Biochem. 215, 109002. doi: 10.1016/j.plaphy.2024.109002, PMID: 39106767

[B27] Jabeen S. Ali M. F. Mohi ud Din A. Javed T. Mohammed N. S. Chaudhari S. K. . (2023). Phytochemical screening and allelopathic potential of phytoextracts of three invasive grass species. Sci. Rep. 13, 8080. doi: 10.1038/s41598-023-35253-x, PMID: 37202455 PMC10195856

[B28] Jabeen S. Mukhtar A. Hussain S. Hussain S. Hussan M. U. Ahmad M. . (2025). Silver nanoparticles mitigated cadmium toxicity in tobacco by modulating biochemical, cellular and genetic responses. Environ. Science: Nano 12, 4081–4095. doi: 10.1039/D5EN00220F

[B29] Kabra K. Chaudhary R. Sawhney R. L. (2004). Treatment of hazardous organic and inorganic compounds through aqueous-phase photocatalysis: a review. Ind. Eng. Chem. Res. 43, 7683–7696. doi: 10.1021/ie0498551

[B30] Kaleem Z. Shahbaz H. Ali S. Albert A. He D. Gulzar R. M. A. . (2025). Melatonin and nanocopper synergistically regulate cadmium toxicity in Brassica napus: evidences from photosynthesis phenomics, oxidative metabolism, and multiple defense responses. Environ. Science: Nano. 12, 3714–3730. doi: 10.1039/D5EN00012B

[B31] Konate A. He X. Zhang Z. Ma Y. Zhang P. Alugongo G. M. . (2017). Magnetic (Fe3O4) nanoparticles reduce heavy metals uptake and mitigate their toxicity in wheat seedling. Sustainability 9, 790. doi: 10.3390/su9050790

[B32] Lan X.-Y. Yan Y.-Y. Yang B. Li X.-Y. Xu F.-L. (2019). Subcellular distribution of cadmium in a novel potential aquatic hyperaccumulator–Microsorum pteropus. Environ. pollut. 248, 1020–1027. doi: 10.1016/j.envpol.2019.01.123, PMID: 31091634

[B33] Lee J. Lee J.-H. Lee S.-Y. Park S. A. Kim J. H. Hwang D. . (2023). Antioxidant iron oxide nanoparticles: their biocompatibility and bioactive properties. Int. J. Mol. Sci. 24, 15901. doi: 10.3390/ijms242115901, PMID: 37958885 PMC10649306

[B34] Liu D. Kottke I. (2003). Subcellular localization of Cd in the root cells of Allium sativum by electron energy loss spectroscopy. J. Biosci. 28, 471–478. doi: 10.1007/BF02705121, PMID: 12799493

[B35] Liu J. Ni J. Mo A. Fan X. Jiang Y. Xie H. . (2023). Cadmium affects the growth, antioxidant capacity, chlorophyll content, and homeostasis of essential elements in soybean plants. South Afr. J. Bot. 162, 604–610. doi: 10.1016/j.sajb.2023.09.059

[B36] Long J. Wang X. Zhang W. (2024). Combined toxicity of multiwall carbon nanotubes and cadmium on rice (Oryza sativa L.) growth in soil. Front. Environ. Sci. 12, 1469172. doi: 10.3389/fenvs.2024.1469172

[B37] Lou L. Li X. Chen J. Li Y. Tang Y. Lv J. (2018). Photosynthetic and ascorbate-glutathione metabolism in the flag leaves as compared to spikes under drought stress of winter wheat (Triticum aestivum L.). PloS One 13, e0194625. doi: 10.1371/journal.pone.0194625, PMID: 29566049 PMC5864061

[B38] Luo P. Wu J. Li T.-T. Shi P. Ma Q. Di D.-W. (2024). An overview of the mechanisms through which plants regulate ROS homeostasis under cadmium stress. Antioxidants 13, 1174. doi: 10.3390/antiox13101174, PMID: 39456428 PMC11505430

[B39] Ma P. Zang J. Shao T. Jiang Q. Li Y. Zhang W. . (2023). Cadmium distribution and transformation in leaf cells involved in detoxification and tolerance in barley. Ecotoxicology Environ. Saf. 249, 114391. doi: 10.1016/j.ecoenv.2022.114391, PMID: 36508843

[B40] Merinero M. Alcudia A. Begines B. Martínez G. Martín-Valero M. J. Pérez-Romero J. A. . (2022). Assessing the biofortification of wheat plants by combining a plant growth-promoting rhizobacterium (PGPR) and polymeric Fe-nanoparticles: allies or enemies? Agronomy 12, 228. doi: 10.3390/agronomy12010228

[B4444] Mozafari A. A. Dedejani S. Ghaderi N. . (2018). Positive responses of strawberry (Fragaria× ananassa Duch.) explants to salicylic and iron nanoparticle application under salinity conditions. Plant Cell Tissue Organ Cult. 134, 267–275. doi: 10.1007/s11240-018-1420-y

[B41] Muhammad F. Daji L. Awan M. I. Mahmood A. Sadaf S. Mukhtar A. . (2024). Combined application of nitrogen and sulfur improves growth, oil, and bio-diesel production from soybean. Polish J. Environ. Stud. 34, 6729–6739. doi: 10.15244/pjoes/192566

[B42] Rao G. P. Lu C. Su F. (2007). Sorption of divalent metal ions from aqueous solution by carbon nanotubes: a review. Separation purification Technol. 58, 224–231. doi: 10.1016/j.seppur.2006.12.006

[B43] Reese R. N. Roberts L. W. (1984). Cadmium uptake and its effects on growth of tobacco cell suspension cultures. Plant Cell Rep. 3, 91–94. doi: 10.1007/BF02441007, PMID: 24253432

[B44] Salam A. Khan A. R. Liu L. Yang S. Azhar W. Ulhassan Z. . (2022). Seed priming with zinc oxide nanoparticles downplayed ultrastructural damage and improved photosynthetic apparatus in maize under cobalt stress. J. Hazardous Materials 423, 127021. doi: 10.1016/j.jhazmat.2021.127021, PMID: 34488098

[B45] Shetty B. R. Jagadeesha P. B. Salmataj S. (2025). Heavy metal contamination and its impact on the food chain: exposure, bioaccumulation, and risk assessment. CyTA-Journal Food 23, 2438726. doi: 10.1080/19476337.2024.2438726

[B46] Sutrisno C. A. P. Kartini K. (2025). Dot-blot assay with AlCl 3 reagent for rapid screening of total flavonoid content in food and herbal medicine materials. Acta Chromatographica. 37, 498–508. doi: 10.1556/1326.2025.01298

[B47] Van Belleghem F. Cuypers A. Semane B. Smeets K. Vangronsveld J. d’Haen J. . (2007). Subcellular localization of cadmium in roots and leaves of Arabidopsis thaliana. New Phytol. 173, 495–508. doi: 10.1111/j.1469-8137.2006.01940.x, PMID: 17244044

[B49] Wang P. Chen H. Kopittke P. M. Zhao F.-J. (2019). Cadmium contamination in agricultural soils of China and the impact on food safety. Environ. pollut. 249, 1038–1048. doi: 10.1016/j.envpol.2019.03.063, PMID: 31146310

[B48] Wang M. Luo J. Li H. Ge C. Jing F. Guo J. . (2025). Synergistic effect of foliar exposure to TiO 2 nanoparticles and planting density modulates the metabolite profile and transcription to alleviate cadmium induced phytotoxicity to wheat (Triticum aestivum L.). Environ. Science: Nano 12, 879–893. doi: 10.1039/D4EN00763H

[B50] Wang Y. Wang Y. Tang Y. Zhu X.-G. (2022). Stomata conductance as a goalkeeper for increased photosynthetic efficiency. Curr. Opin. Plant Biol. 70, 102310. doi: 10.1016/j.pbi.2022.102310, PMID: 36376162

[B51] Wu L. Ma L. Pan W. Zhang Y. Tian Y. Zhu Y. . (2024). Optimal antioxidant enzyme activity estimation in melon plant leaves based on microhyperspectral imaging technique. Microchemical J. 206, 111626. doi: 10.1016/j.microc.2024.111626

[B52] Xin X. Zhao F. Rho J. Y. Goodrich S. L. Sumerlin B. S. He Z. (2020). Use of polymeric nanoparticles to improve seed germination and plant growth under copper stress. Sci. Total Environ. 745, 141055. doi: 10.1016/j.scitotenv.2020.141055, PMID: 32736110

[B54] Yang X. Alidoust D. Wang C. (2020). Effects of iron oxide nanoparticles on the mineral composition and growth of soybean (Glycine max L.) plants. Acta Physiologiae Plantarum 42, 128. doi: 10.1007/s11738-020-03104-1

[B53] Yang Q. Li Z. Lu X. Duan Q. Huang L. Bi J. (2018). A review of soil heavy metal pollution from industrial and agricultural regions in China: Pollution and risk assessment. Sci. total Environ. 642, 690–700. doi: 10.1016/j.scitotenv.2018.06.068, PMID: 29909337

[B55] Yasin M. U. Haider Z. Munir R. Zulfiqar U. Rehman M. Javaid M. H. . (2024). The synergistic potential of biochar and nanoparticles in phytoremediation and enhancing cadmium tolerance in plants. Chemosphere 354, 141672. doi: 10.1016/j.chemosphere.2024.141672, PMID: 38479680

[B56] Younas M. Nafees M. Munir M. Alomrani S. O. Waseem M. Alshehri M. A. . (2025). Cadmium resistance microbes and TiO2 nanoparticles alleviate cadmium toxicity in wheat. Sci. Rep. 15, 5557. doi: 10.1038/s41598-025-88371-z, PMID: 39953062 PMC11829019

[B57] Yu J.a. Chen Z. Gao W. He S. Xiao D. Fan W. . (2025). Global trends and prospects in research on heavy metal pollution at contaminated sites. J. Environ. Manage. 383, 125402. doi: 10.1016/j.jenvman.2025.125402, PMID: 40262497

[B58] Yuan H. M. Huang X. (2016). Inhibition of root meristem growth by cadmium involves nitric oxide-mediated repression of auxin accumulation and signalling in Arabidopsis. Plant Cell Environ. 39, 120–135. doi: 10.1111/pce.12597, PMID: 26138870

[B59] Zhang S. Song J. Wu L. Chen Z. (2021). Worldwide cadmium accumulation in soybean grains and feasibility of food production on contaminated calcareous soils. Environ. pollut. 269, 116153. doi: 10.1016/j.envpol.2020.116153, PMID: 33309406

[B60] Zhao D. Lin G.-B. Liu C. Juhasz A. L. Ma L. Q. (2025). Health risk assessment of dietary cadmium exposure based on cadmium bioavailability in food: Opportunities and challenges. J. Hazardous Materials 488, 137359. doi: 10.1016/j.jhazmat.2025.137359, PMID: 39874772

[B61] Zhou P. Adeel M. Shakoor N. Guo M. Hao Y. Azeem I. . (2020). Application of nanoparticles alleviates heavy metals stress and promotes plant growth: An overview. Nanomaterials 11, 26. doi: 10.3390/nano11010026, PMID: 33374410 PMC7824443

[B62] Zhou P. Zhang P. He M. Cao Y. Adeel M. Shakoor N. . (2023). Iron-based nanomaterials reduce cadmium toxicity in rice (Oryza sativa L.) by modulating phytohormones, phytochelatin, cadmium transport genes and iron plaque formation. Environ. pollut. 320, 121063. doi: 10.1016/j.envpol.2023.121063, PMID: 36639045

[B63] Zia-ur-Rehman M. Mfarrej M. F. B. Usman M. Anayatullah S. Rizwan M. Alharby H. F. . (2023). Effect of iron nanoparticles and conventional sources of Fe on growth, physiology and nutrient accumulation in wheat plants grown on normal and salt-affected soils. J. Hazardous Materials 458, 131861. doi: 10.1016/j.jhazmat.2023.131861, PMID: 37336110

