# Supplementary material for: Iron nanoparticles mitigate cadmium-induced abiotic stress in soybean by modulating reactive oxygen species accumulation and cellular integrity
Source: Front Plant Sci. 2026 Jan 16;16:1727507. doi: 10.3389/fpls.2025.1727507 (PMC12856577; doi:10.3389/fpls.2025.1727507)

[1(1).raw]	Peak Search Report

SCAN: 5.0/80.0003/0.01966/23.04(sec), Fe, I(max)=442, 07/18/24 14:11

PEAK: 19-pts/Parabolic Filter, Threshold=3.0, Cutoff=0.1%, BG=3/1.0, Peak-Top=Summit

NOTE: Intensity = Counts, 2T(0)=0.0(deg), Wavelength to Compute d-Spacing = 1.93604Å (Fe/K-alpha1)

#	2-Theta	d(Å)	(h k l)	BG	Height	Height%	Area	Area%	FWHM
1	24.133	4.6307		94	86	32.6	13.8	18.3	0.136
2	33.154	3.3930		178	264	100.0	75.2	100.0	0.242
3	35.631	3.1639		167	201	76.1	56.7	75.4	0.240
4	40.894	2.7710		168	103	39.0	20.4	27.1	0.168
5	49.432	2.3152		108	103	39.0	38.4	51.0	0.317
6	54.033	2.1310		99	127	48.1	44.0	58.5	0.294
7	57.640	2.0081		90	58	22.0	7.9	10.5	0.116
8	62.408	1.8684		78	98	37.1	38.1	50.7	0.331
9	62.599	1.8633		79	61	23.1	37.7	50.1	0.525
10	64.018	1.8263		82	82	31.1	26.6	35.3	0.275
11	71.911	1.6487		69	44	16.7	16.0	21.3	0.310

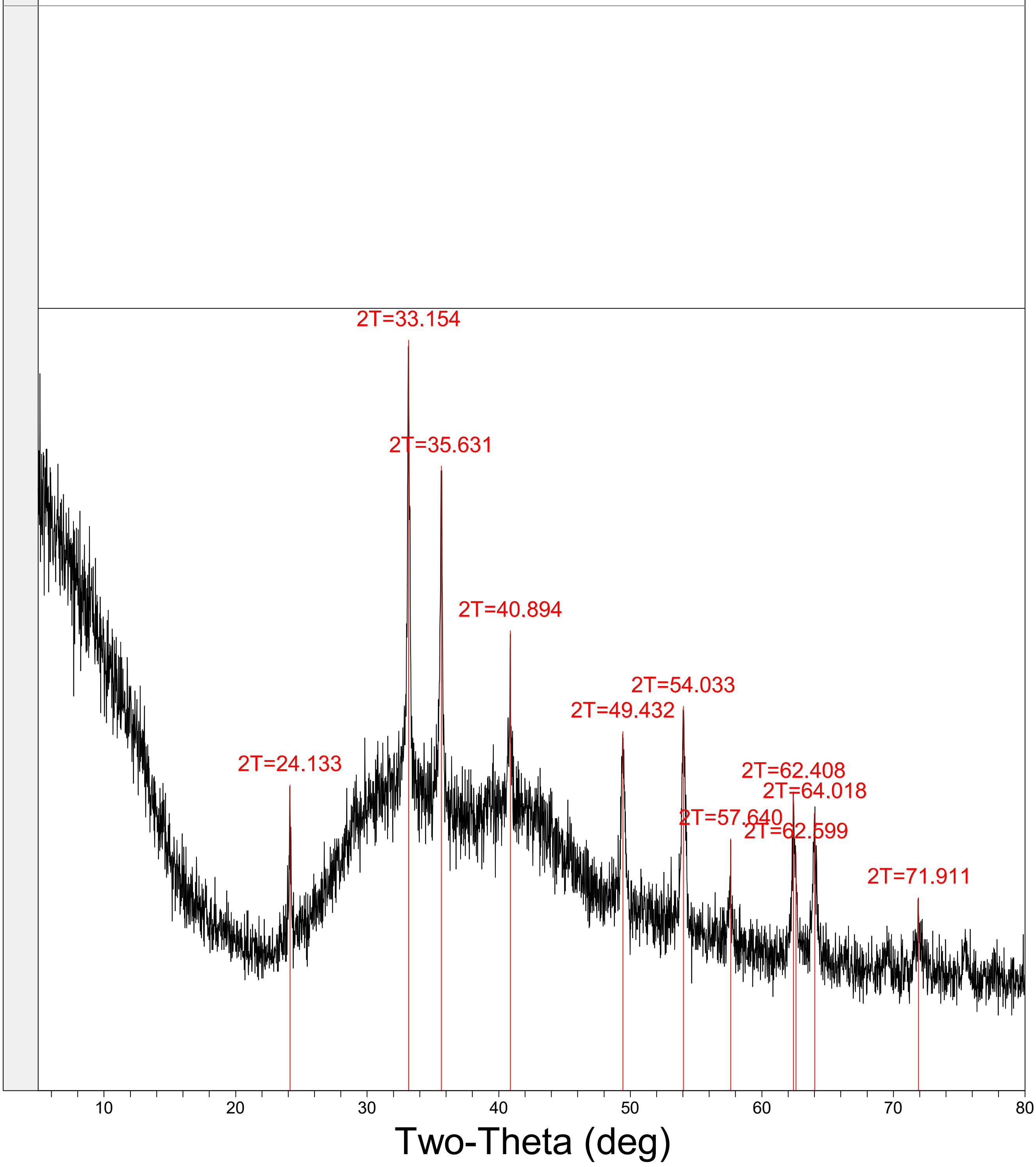

Supplement: Supplementary file 1 [file DataSheet1.pdf]
